# Study on blasting characteristics of rock mass with weak interlayer based on energy field

**DOI:** 10.1038/s41598-022-17028-y

**Published:** 2022-07-26

**Authors:** Jianbin Cui, Liangfu Xie, Wei Qiao, Liewang Qiu, Zeyu Hu, Liming Wu

**Affiliations:** 1 College of Civil Engineering and Architecture, Xinjiang University, Urumqi, 830017 China; 2 Xinjiang Civil Engineering Technology Research Center, Urumqi, 830017 China; 3 Xinjiang Academy of Architectural Science (Limited Liability Company), Urumqi, 83002 China

**Keywords:** Civil engineering, Petrology

## Abstract

In order to explore the influence of weak interlayer on blasting characteristics in natural rock mass, by using the particle flow code (PFC^2D^), a single hole blasting numerical model of hard rock with soft interlayer is established. The blasting experiments at different positions and thicknesses of weak interlayer are carried out. Then an in-depth analysis from the perspectives of crack effect, stress field and energy field is made. Results showed that: (i) When the explosion is initiated outside the weak interlayer, if the interlayer is located within about twice the radius of the crushing area, the closer the interlayer is to the blast hole, the higher the damage degree of the rock mass around the blast hole. And the number of cracks will increase by about 1–2% when the distance between the weak interlayer and the blast hole decreases by 0.5 m. (ii) When detonating outside the weak interlayer, if the interlayer is within about 4 times radius of the crushing area, the hard rock on both sides of the weak interlayer will in a high stress state. Under the same case, the peak kinetic energy and peak friction energy will increase by about 23 and 13%, respectively, and the peak strain energy will increase by about 218 kJ for every 0.1 m increase in the thickness of the weak interlayer.

## Introduction

In engineering geology, interlayer often refers to the layered rock mass with a certain thickness mixed in the rock mass, which may be fault fracture zone and argillaceous interlayer. Its mechanical properties are often significantly different from those of surrounding rock^1^. In addition, the weak interlayer also seriously hinders the propagation of stress wave and intensifies the attenuation of stress wave energy^2^. In addition to this kind of layered rock mass, there are a large number of structural planes such as joints, fissures and weak interlayer in natural rock mass. Their mechanical properties are quite different from the surrounding rock mass, and they also seriously affect the propagation of stress wave^3–9^.

With the continuous application of blasting technology in underground engineering^10^, ore mining^11–13^ and other geological engineering, the blasting experiment of natural rock mass with structural plane is also the research hotspot of many scholars. The existence of faults will have a serious impact on the generation and development of cracks after blasting, as well as on the propagation of stress wave^14^. When the stress wave passes through the fault, part of the energy of the stress wave will be absorbed by the fault, resulting in obvious attenuation of dynamic response, and the angle of the fault has a great impact on this phenomenon^15^. During the propagation of stress wave, due to the existence of fault, the reflection effect of stress wave will also occur^16^, which intensifies the damage of rock mass in the area between fault and fault^17,18^. The structure of weak interlayer is similar to that of fault. During blasting construction, weak interlayer is a weak link in rock mass engineering, which often has adverse effects on the project^19–22^. Wang et al. proposed a dynamic test method of elastic modulus of weak interlayer for one-dimensional rock mass with weak interlayer^23^. Combined with the measured data, Duan et al. put forward a comprehensive scheme for in-situ observation of rock mass in soft interlayer zone of large underground cavern, so as to observe the development process of rock mass failure^24^. Song et al. designed and developed a large-scale three-dimensional tunnel excavation simulation test system, and found that the blasting process of tunnel backward excavation has a great impact on the stability of intercalated rock mass^25^. Zhang et al. put forward the concept of critical explosion energy and believed that when the sum of explosion energy produced by various factors is less than the critical explosion energy, the dynamic response is mainly affected by the internal structure of the slope^26^. Man et al. found that the instability of the tunnel face is significantly aggravated by the weak interlayer, and the position and thickness of the weak interlayer also have a certain impact on the stability of the tunnel face^27^.

If the initiation position is near the weak interlayer, the results after blasting may be quite different from the original plan, which makes the blasting effect difficult to control. For the problem of weak interlayer in natural rock mass, most scholars consider it from the macro perspective of theoretical analysis or engineering, and few scholars can make in-depth analysis from the perspective of local failure characteristics of rock mass. Therefore, in order to make the blasting effect more controllable and supplement the research on the blasting of rock mass with weak interlayer, based on the discrete element software, a blasting numerical model of rock mass with weak interlayer is established in this paper. Then, the blasting characteristics are analyzed from the perspectives of crack effect, stress field and energy field. The conclusions can provide reference for practical blasting engineering.

## Verification of single hole blasting experiment

### Stress wave propagation law

When the stress wave propagates from one medium to another, the transmission, reflection and refraction of the stress wave will occur. Part of the stress wave is reflected at the interface and continues to propagate in the original medium, while the other part passes through the interface and enters another medium. According to different incident angles, the incidence of stress wave can be divided into two cases: vertical incidence and inclined incidence. Because the situation of inclined incidence of stress wave is very complex, longitudinal wave and transverse wave will be generated again after the incident wave propagates to the structural plane. Therefore, only the case of vertical incidence of stress wave is discussed here. When the stress wave is vertically incident through the structural plane, there will only be transmission and reflection. The calculation formulas of transmitted stress wave and reflected stress wave are as follows^28^:

1 $$\begin{gathered} \sigma_{r} = R\sigma_{i} \hfill \\ \sigma_{t} = T\sigma_{i} \hfill \\ \end{gathered}$$

where *σ*_*i*_, *σ*_*t*_, and *σ*_*r*_ are the incident stress wave, transmitted stress wave and reflected stress wave. The *R* and *T* are the reflected coefficient and transmitted coefficient, and they can expressed by formula (2)^28^:

2 $$\begin{gathered} R = (1 - n)/(1 + n) \hfill \\ T = 2/(1 + n) \hfill \\ \end{gathered}$$

where *n* = *ρ*_1_*c*_1_/*ρ*_2_*c*_2_ is the acoustic impedance ratio, the *ρ*_1_ and *ρ*_2_ are the density of the first material and the second material, the *c*_1_ and *c*_2_ are the stress wave velocity of the first material and the second material.

### Select model parameters

**Figure 1:**
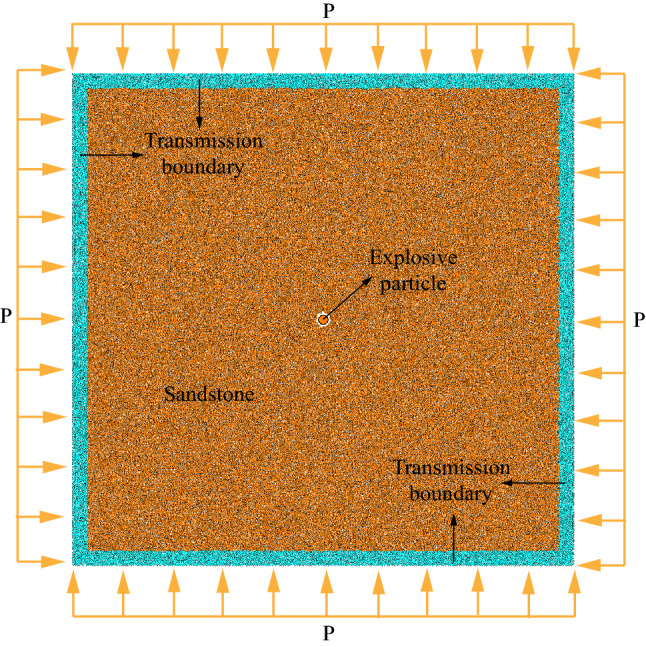
Schematic diagram of single hole blasting model.

Based on particle flow code (PFC^2D^), the single hole blasting numerical model (Fig. 1) is set up, which scale is 10 m × 10 m. Additionally, the initial radius range of the model is 5 ~ 7.5 mm before generated by the way of particle expansion. The explosion point is simplified to a particle with 10 cm diameter which is set in the center of the model. In order to simulate the real rock, the parallel bond model (PBM) in PFC^2D^ is selected for the model. The difference between parallel bond model (PBM) and other models is mainly reflected in the different contacts between particles. When the contact of PBM is bonded, it can transfer normal force, tangential force and moment. After the contact is unbonded, it degenerates into the linear model, which can only transfer normal force. Therefore, this model is widely used to simulate rock and soil materials. The initial stress state (isotropic compressive pressure of P = 5 MPa) of this model is achieved through the servo mechanism proposed by Cundall et al.^29^.

This paper takes sandstone as the research object which is tested well by Wei Yuan et al. based on single blasting numerical simulation. Therefore, the microscopic parameters of sandstone obtained by Wei Yuan et al. are used in this paper (Table 1)^30^.

**Table 1:** Microscopic parameters of sandstone.

Linear group	Parallel-bond group
Effective modulus = 51.0 GPa	Bond effective modulus = 42.0 GPa
Friction coefficient = 1.0	Bond stiffness ratio = 1.0
Stiffness ratio = 1.0	Bond tensile strength = 30.0 MPa
	Bond cohesion = 350.0 MPa
	Bond friction = 65°

**Figure 2:**
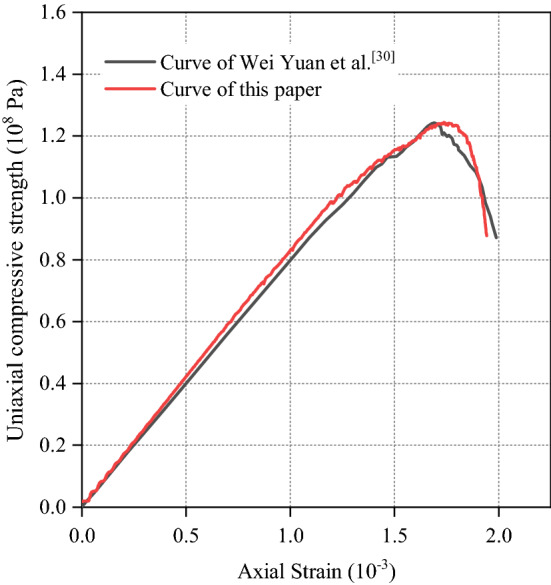
Comparison of uniaxial compression test results.

**Figure 3:**
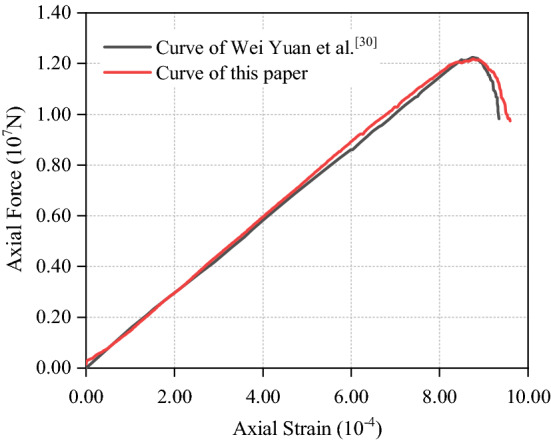
Comparison of Brazilian splitting test results.

**Figure 4:**
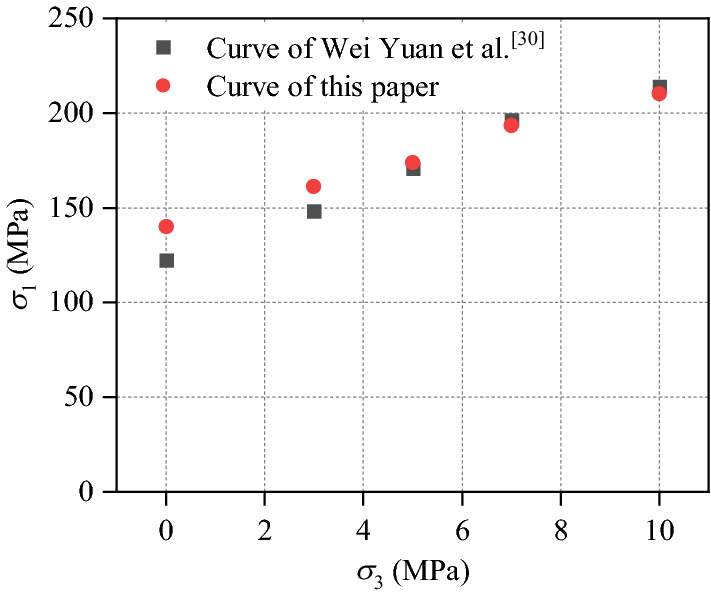
Comparison of biaxial compression test.

In order to verify the rationality of selecting parameters (Table 1), the uniaxial compression experiment, Brazilian splitting test and biaxial compression test are carried out. The specimen size of uniaxial compression test and biaxial compression test is 50 mm × 100 mm, and the specimen shape of Brazilian splitting test is a circle with a diameter of 0.9 m. What’s more, the particle radius range, particle formation mode and constitutive model in all tests are the same as those of the validation experimental model. The obtained stress–strain curves are shown in Figs. 2, 3 and 4, which shows that the parameters in Table 1 are reasonable and accurate.

### Set boundary conditions

In order to make stress wave not reflect from the boundary, this paper considers the viscous boundary proposed by Kouroussis and Verlinden^31^ and the dispersion effect of stress wave propagation at rock mass boundary proposed by Shi^32^.

The relationship between boundary force and particle moving velocity is:

3 $$F = - 2\rho C\dot{u}r$$

where *r* is the particle radius, *ρ* is the rock density, *C* is the wave velocity, $\dot{u}$ is the particle velocity.

4 $$F{ = }\left\{ {\begin{array}{*{20}c} { - \xi \, \cdot \, 2\rho C_{{\text{P}}} \dot{u}_{{\text{n}}} r} \\ { - \eta \, \cdot \, 2\rho C_{{\text{s}}} \dot{u}_{{\text{s}}} r} \\ \end{array} } \right.$$

where *ζ* and *η* are the dispersion effect correction coefficients of P-wave and S-wave respectively; *C*_P_ and *C*_S_ are P-wave velocity and S-wave velocity respectively; $\dot{u}_{{\text{n}}}$ and $\dot{u}_{{\text{s}}}$ are the normal and tangential velocities of particles respectively.

### Apply the blasting load

Blasting point expansion method is selected to apply blasting load^32^. When the explosion point particle expands, it will overlap with the particles of the surrounding rock mass. According to the particle contact principle of PFC, the radial force *F* on the surrounding rock particles after explosion point expansion is:

5 $$F = K_{{\text{n}}} d = 2{\uppi }r_{{0}} p$$

Then the explosion point particle expansion radius is:

6 $$d = \frac{{2{\uppi }r_{{0}} p}}{{K_{{\text{n}}} }}$$

where *K*_n_ is the contact stiffness of particles, *r*_0_ is the initial radius of the blast point, *d*is the blast point radius after expansion, *p* is the stress acting on the rock wall.

**Figure 5:**
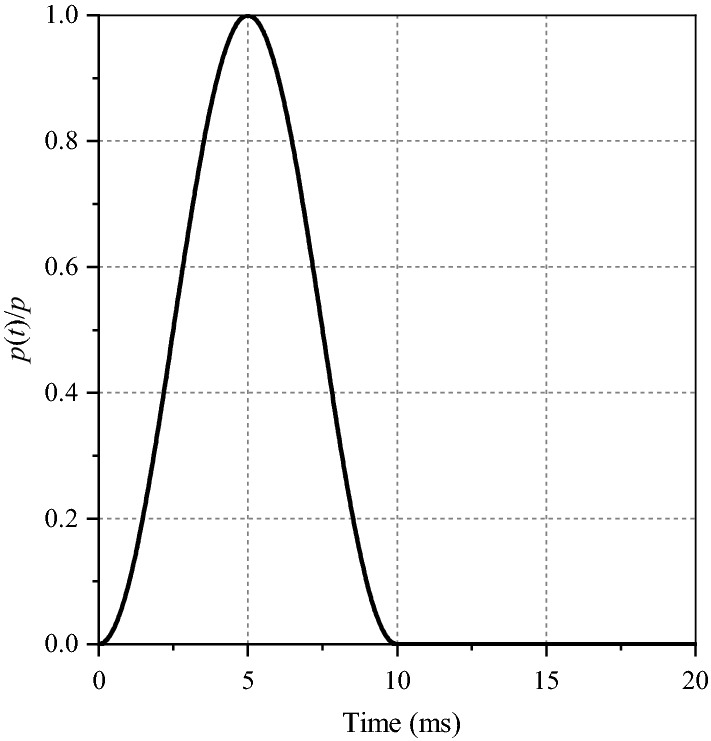
Blast pressure load time history curve.

The explosion load propagates to the surrounding rock mass with the explosion point as the center, and the action form is equivalent to pulse wave. It is simplified as a half sine wave (also known as time history curve), as shown in Fig. 5 with the same time in the rising section and the falling section, and its expression is:

7 $$p(t){ = }\frac{p}{2}\left( {1 - \cos \left( {\frac{{2{\uppi }}}{\Delta T}t} \right)} \right)$$

where *p*(*t*) is the blasting load on the hole wall, *p* is the peak pressure in the hole, △*T* is the half sinusoidal action time, generally 10 ms, and *t* is the duration, which is 20 ms.

### Verify the simulation results

**Figure 6:**
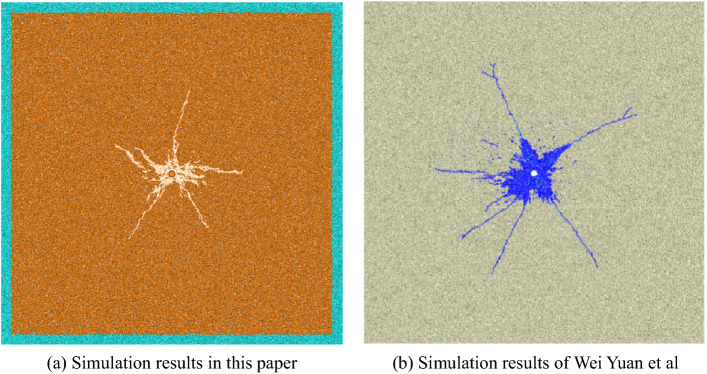
Numerical simulation in this paper (**a**) and numerical simulation by Wei Yuan et al. (**b**)^30^.

Final blasting effect is shown in Fig. 6a. Under the same model size, rock mechanical properties, and initial stress field, the result is basically consistent with that obtained by Wei Yuan et al.^30^ (Fig. 6b), which proves the accuracy of the blasting method adopted in this paper.

## Working cases setting

In the actual blasting engineering, weak interlayer is often encountered. The position and thickness of interlayer often have a great impact on the blasting quality and effect. Based on this, this paper carries out single hole blasting experiments at different positions and thicknesses of weak interlayer.

Based on the verification experiment of the above hard rock, some soft rocks are introduced as soft interlayer. The specific distribution cases of soft interlayer are shown in Table 2. Xie et al.^33^ used PFC^2D^ to conduct uniaxial compression test and biaxial compression test, accurately calibrated the micro parameters of limestone (Table 3), and took this group of micro parameters as the parameters of soft rock in this paper.

**Table 2:** Distribution case of weak interlayer.

Distance between blast hole and interlayer		Thickness of interlayer		Working diagram
Cases	H (m)	Cases	H_S_ (m)	
1	0.0	1	0.1	
2	0.5	2	0.2	
3	1.0	3	0.3	
4	1.5	4	0.4	
5	2.0	5	0.5	
6	2.5			
7	3.0			
8	3.5

**Table 3:** Microscopic parameters of limestone.

Linear group	Parallel-bond group
Effective modulus = 2.5 GPa	Bond effective modulus = 2.5 GPa
Friction coefficient = 0.2	Bond stiffness ratio = 1.8
Stiffness ratio = 1.8	Bond tensile strength = 10.0 MPa
	Bond cohesion = 5.0 MPa
	Bond friction = 10°

## Analysis of blasting results

### Blasting crack states

**Figure 7:**
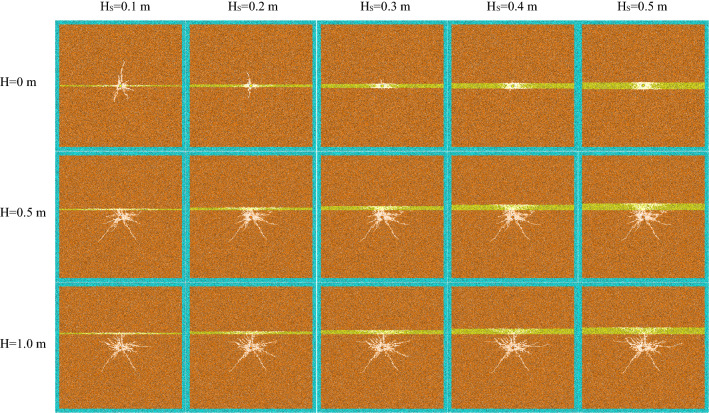
The strengthening section of crack development.

**Figure 8:**
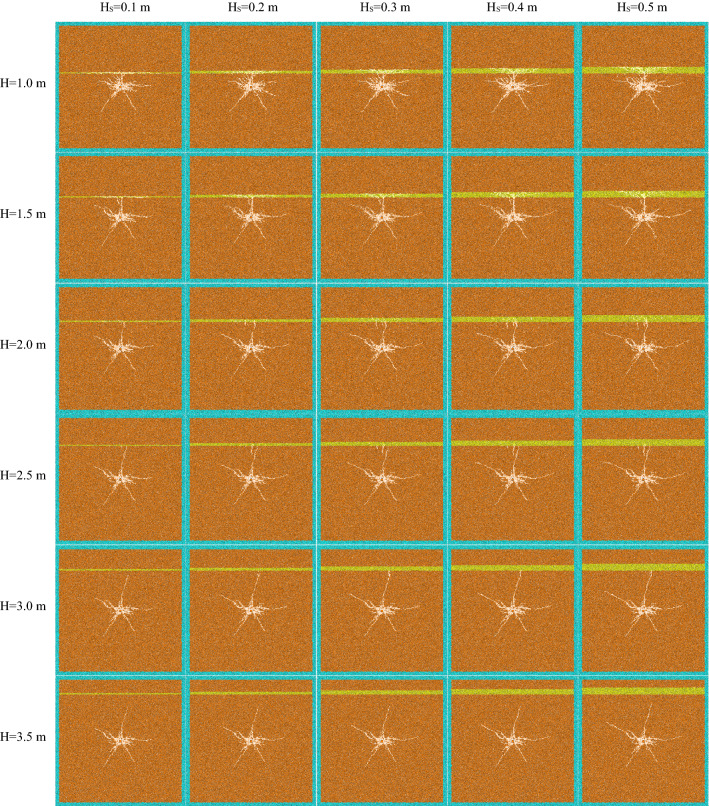
The declining section of crack development.

The experimental results are divided into two parts according to the characteristics of crack development, the strengthening section of crack development (Fig. 7) and the declining section of crack development (Fig. 8).

When the thickness of weak interlayer (H_S_) is constant and the distance from blast hole H < 1.0 m (about twice the radius of crushing area), the length and number of cracks increase significantly with the increase of H, and the crack shape changes greatly. When the weak interlayer is on one side of the blast hole, with the increase of the distance between the interlayer and the blast hole, the crack change in the soft rock is mainly manifested in the extension of the crack in the transverse direction (parallel to the direction of the weak interlayer) and the increase of the crushing area. Due to the large consumption of stress wave in soft rock, the hard rock on the upper side of soft interlayer is hardly affected, which also shows that weak interlayer can quickly and greatly reduce the energy generated by blasting, so as to block the propagation of stress wave. In hard rock, the change of crack is mainly reflected in the increase of rock damage degree of blast hole and weak interlayer. Due to the stress wave reflected by structural plane and the blocking effect of crack on stress wave, the superposition of the two causes the further development of main crack, while the hard rock foundation below blast hole is not affected by weak interlayer. When the blast hole is located in the weak interlayer, because the soft rock can consume more energy generated by blasting, the energy transmitted into the hard rock is greatly reduced, resulting in the reduction of crack propagation range.

When the distance (H) between the weak interlayer and the blast hole is certain and the interlayer is on one side of the blast hole, the number of cracks increases and the final state changes with the increase of the thickness (H_S_) of the weak interlayer. In the hard rock, the number of cracks has increased significantly, the expansion range has also increased, and the damage degree of the rock mass around the blast hole is also increasing, indicating that the thicker the weak interlayer, the more reflected stress waves, resulting in more serious damage to the hard rock of the blast hole and the weak interlayer. In soft rock, with the increase of H_S_, it can be seen that the crack propagation range is significantly reduced. When the blast hole is located in the weak interlayer, the crack range is also significantly reduced with the increase of H_S_. At the same time, the number and length of cracks in the hard rock on the upper and lower sides are also significantly reduced. It shows that the thin soft rock interlayer can not consume the stress wave generated by blasting, so that the stress wave is transmitted to the hard rock on the upper and lower sides, resulting in the cracking of hard rock. With the increase of the thickness of soft rock interlayer, the consumed stress wave will also increase significantly, resulting in a significant reduction of cracks in the hard rock parts on the upper and lower sides. When H_S_ > 0.3 m, most of the energy generated by blasting can be consumed by the soft rock interlayer, resulting in almost no cracks in the hard rock on both sides. At the same time, with the increase of H_S_, the damage degree of rock mass around blast hole is obviously improved.

When the thickness of weak interlayer is constant and the distance from blast hole H ≥ 1.0 m, the number of cracks decreases with the increase of H, but the crack propagation range changes little. It shows that when the weak interlayer is outside the radius of 2 times the crushing area, with the increase of H, the reflected stress wave is decreasing, and the energy in some rocks converging between the blast hole and the structural plane is also decreasing. Therefore, the main crack cannot continue to extend, and the damage degree of the rocks around the blast hole is weakened. With the increase of H, the cracks in the soft rock have been greatly attenuated when the stress wave diffuses to the interlayer due to the long distance, which greatly reduces the number and propagation length of cracks in the weak interlayer. When the distance (H) between the weak interlayer and the blast hole is constant, with the increase of H_S_, the conclusion is basically the same as that in the rising section of the number of cracks.

### Blasting crack number

**Figure 9:**
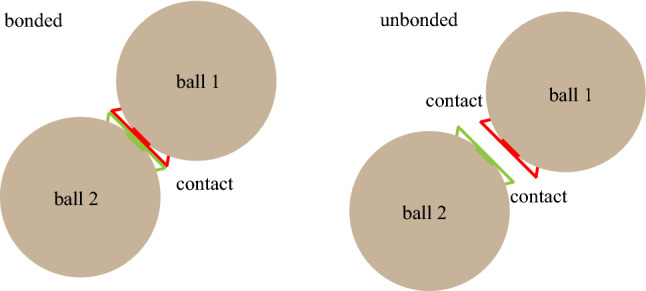
Schematic diagram of particle and contact.

Particle and contact are two basic elements of numerical model in the particle flow code (PFC^2D^). The contact between particles can transfer load (Fig. 9), thus simulating the mechanical properties of rock. Once the local strength limit is exceeded, the contact will break, the two particles will separate from each other, and the program will determine that there is a crack between the two particles. In this paper, the parallel bond model (PBM) is adopted, and the failure conditions of the model are shown in formula (8). If the tensile stress ($\sigma_{c}$) exceeds the tensile strength ($\sigma_{c}$ > $\sigma_{t\max }$) or the shear stress ($\tau_{c}$) exceeds the shear strength ($\tau_{c}$ > $\tau_{\max }$), it means that the bonded interface breaks, leading to the formation of the new crack. The number of particles and contacts of the whole rock sample are certain. The more the number of cracks, the higher the damage degree of the rock in a certain range. Therefore, the statistical change of the number of cracks can also reflect the severity of rock failure in a certain range.

8 $$\left\{ {\begin{array}{*{20}c} {\sigma_{t\max } = \frac{{\overline{{F_{n} }} }}{A} + \frac{{\left| {\overline{M} } \right|}}{I}\overline{R} } \\ {\tau_{\max } = \frac{{\overline{{F_{S} }} }}{A}} \\ \end{array} } \right.$$

where $\sigma_{t\max }$ and $\tau_{\max }$ represent the maximum normal stress and the maximum shear stress, respectively. $\overline{{F_{n} }}$ and $\overline{{F_{S} }}$ represent the normal and tangential component of the parallel-bonded force ($\overline{F}$), respectively. *A* and *I* are the area and inertial moment of the bond cross section, respectively. $\overline{R}$ is the bond radius.

**Figure 10:**
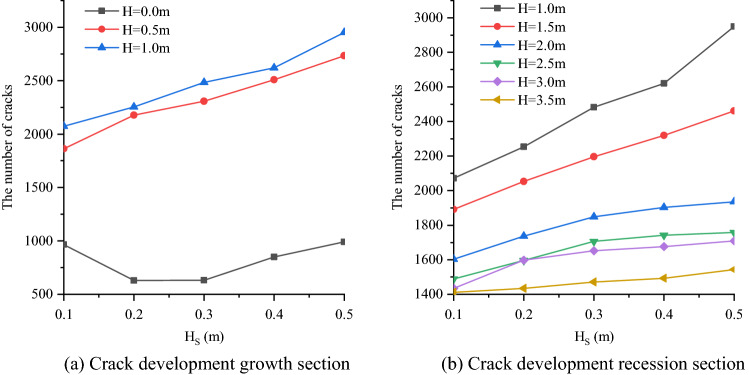
Change curve of crack number.

According to the experimental results, the evolution curve of crack number can be obtained under the cases of different thickness of weak interlayer (H_S_) and different distance between weak interlayer and blast hole (H) (Fig. 10).

The same as the crack development form, according to the distance between the weak interlayer and the blast hole, the change curve of the specific number of cracks can be divided into two parts: the crack number growth section and the crack number decline section.

When the blast hole is in the weak interlayer, the number of cracks decreases first and then increases with the increase of H_S_. When the distance between the weak interlayer and the blast hole (H) is certain and the interlayer is on one side of the blast hole, the number of cracks increases uniformly with the increase of the thickness of the weak interlayer (H_S_). In this case, the change regularity of the number of cracks is strong, and the specific changes are analyzed quantitatively (Table 4). As shown in Table 4, the average value of crack change (i.e. the average value of crack number growth value for each 0.1 m increase of H_S_ in the range of 0.1–0.5 m) will decrease with the increase of H. At the same time, when H is 3.5–0.5 m, the number of cracks will increase by about 1–2% with each 0.5 m decrease in H.

**Table 4:** Change value of crack number.

	H (m)						
	0.5	1.0	1.5	2.0	2.5	3.0	3.5
Average number of growing cracks	218	220	143	83	67	69	33
Average crack growth percentage (%)	9	8	6	5	4	4	2

### Microscopic contact after blasting

**Figure 11:**
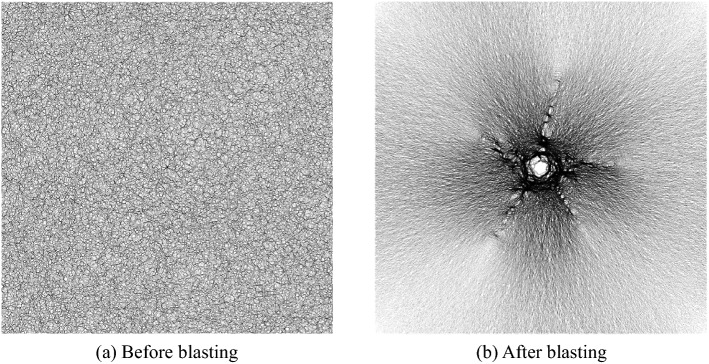
Schematic diagram of contact force distribution.

In PFC^2D^, the contact force between particles can be observed. As shown in Fig. 11, the distribution of contact force under 5 MPa pure hard rock (Fig. 11a) before blasting and Fig. 11b after blasting). In Fig. 11b, the relative magnitude and direction of the contact force between all particles of the rock sample can be clearly seen. The darker the color of the contact force, the greater the contact force, and the lighter the color, the smaller the contact force. The color of the contact force around the blast hole is the darkest, indicating that the contact force around the blast hole is the largest. The color of the tip area of the main crack is very light, and the direction of the contact force is almost perpendicular to the extension direction of the main crack. And the contact force on both sides of the main crack tends to be tensioned to both sides, which shows that the main crack is affected by the tension on both sides, which also explains that the main crack is mainly tensile failure.

**Figure 12:**
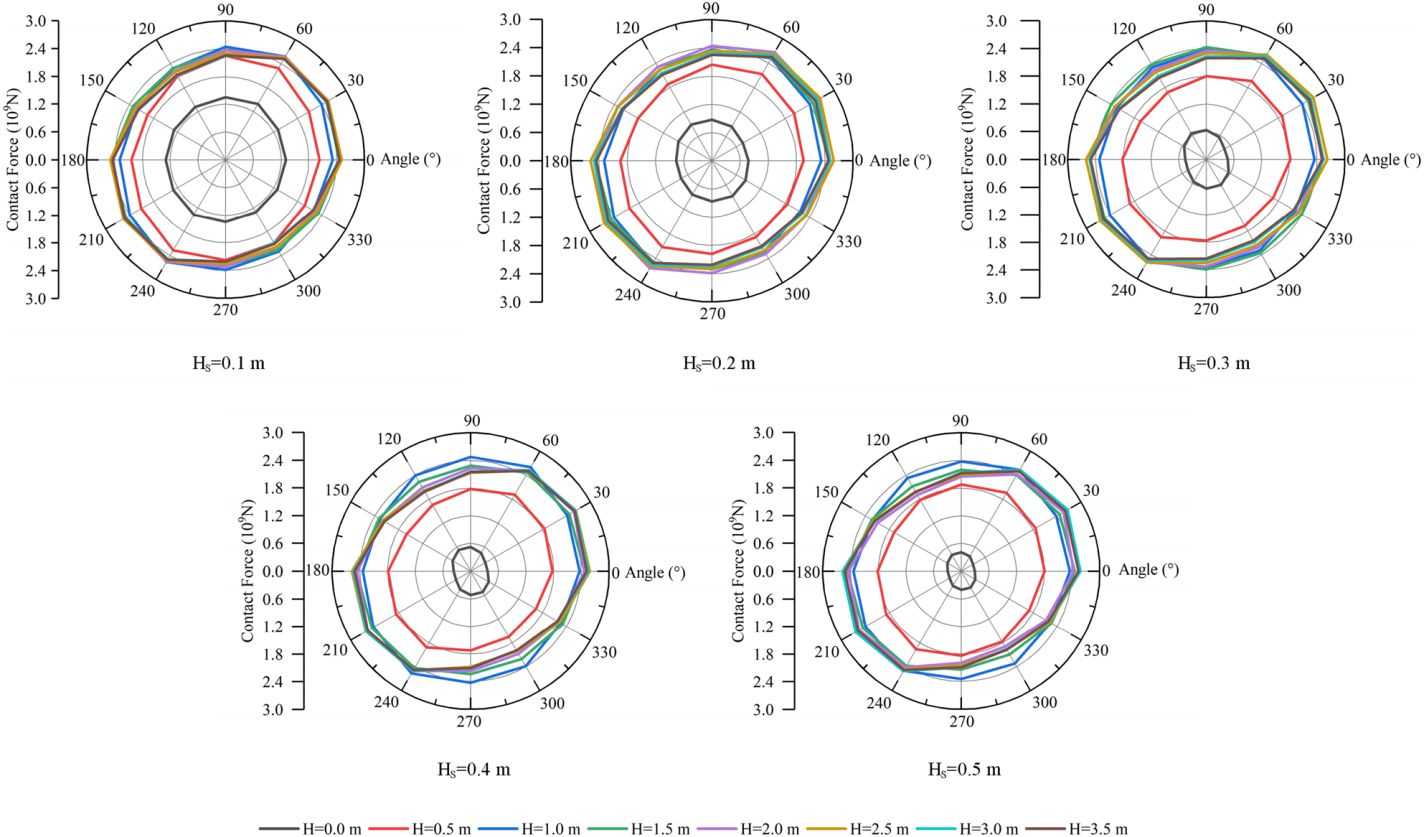
Contact force distribution under different working cases.

Statistics of the magnitude and direction of contact force in rock mass can more intuitively understand the influence of weak interlayer on the overall stress of rock mass samples. Therefore, this paper makes statistics on the contact force information after blasting under all working conditions, and the results are shown in Fig. 12.

When the thickness of weak interlayer (H_S_) is constant, the contact force of rock sample increases with the increase of the distance (H) between blast hole and weak interlayer, especially when H is less than about twice the radius of crushing area (H < 1.0 m). When H is greater than about twice the radius of crushing area (H > 1.0 m), the overall contact force in the rock mass is basically stable and the change is relatively small. This law shows that when the distance between the weak interlayer and the blast hole is less than about twice the radius of the crushing area, the internal stress of the rock mass can be significantly reduced.

When H_S_ = 0.1 m, the distribution of contact force in all directions is relatively uniform, and the curve is basically circular. With the increase of H_S_, the curve is basically elliptical, and the directions of the major axis and minor axis of the elliptic curve are basically unchanged. When H = 0.0 m, it is detonated in soft rock. With the increase of H_S_, the contact force curve shrinks inward in the form of ellipse, and the directions at both ends of the long axis of the ellipse are about 105 and 285° respectively. It shows that when blasting in soft rock, the increase of the thickness of weak interlayer can significantly reduce the internal stress of the whole rock mass, and the main stress directions are 105 and 285°. When H ≠ 0.0 and H is less than twice the radius of the crushing zone, the explosion is initiated in hard rock. With the increase of H_S_, the contact force curve will shrink inward in the form of ellipse, and the directions at both ends of the long axis of the ellipse are about 60 and 240°. The results also show that the increase of the thickness of weak interlayer can significantly reduce the internal stress of the whole rock mass, and the main stress directions are 60 and 240°. When H is greater than twice the radius of the crushing area, it is still detonated in hard rock. At this time, the change of the contact force curve is basically stable and is less affected by H and H_S_. And, the contact force curves are also elliptical, and the directions at both ends of its long axis are about 30 and 210°, indicating that the change of the thickness of weak interlayer has little impact on the internal stress of rock mass.

### Stress variation on both sides of weak interlayer

**Figure 13:**
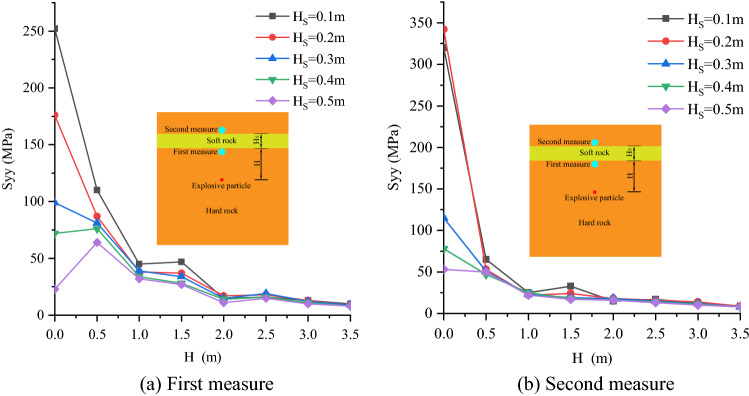
Stress variation curve on both sides of weak interlayer.

In this paper, first measure and second measure point are arranged on the upper and lower sides of the weak interlayer (Fig. 13) to monitor the peak stress Syy. Finally, the variation curve of Syy with the distance between the weak interlayer and the blast hole is obtained (Fig. 13).

As shown in Fig. 13a, when the thickness of weak interlayer (H_S_) is constant, Syy presents two curves of different trends with the increase of the distance between blast hole and structural plane (H):

1. When H_S_ ≤ 0.3 m, Syy shows an overall decreasing trend with the increase of H. When H ≤ 2.0 m, Syy decreases sharply with the increase of H. When H > 2.0 m, the attenuation trend of the curve is slow and the change is relatively stable. When H_S_ is constant, the position of blast hole also changes. When H = 0 m, that is, the blast hole is located in the soft rock. At this time, the Syy of the hard rock on both sides of the soft interlayer is the maximum in the whole curve. Combined with the crack results, Fig. 7 shows that when the soft interlayer is thin (i.e. H_S_ ≤ 0.3 m), the damage of the soft rock caused by stress wave will be transmitted to the hard rock on both sides, making the hard rock in a high stress state. The thinner the soft interlayer, the higher the stress of the rock mass on both sides, and the higher the damage degree of hard rock on both sides. When the weak interlayer is on one side of the blast hole (i.e. H ≠ 0 m) and H_S_ is constant, Syy shows a downward trend with the increase of H. When H ≤ 2.0 m, because the interlayer is close to the blast hole, the stress wave attenuates slowly in the hard rock, and the stress wave reflected by the interlayer is easy to converge in the hard rock between the blast hole and the interlayer, so the stress value of this section of the curve is relatively high. Similarly, when H > 2.0 m, because the interlayer is far from the blast hole, the attenuation amplitude of stress wave is large, and the stress wave reflected by the interlayer is not easy to converge in the hard rock between the blast hole and the interlayer, the stress value of this section of curve is relatively low.
2. When H_S_ > 0.3 m, Syy increases first and then decreases with the increase of H. When H ≤ 0.5 m, Syy increases with the increase of H. When 0.5 < H ≤ 2.0 m, Syy decreases rapidly with the increase of H. When H > 2.0 m, Syy decreases slowly with the increase of H, and the change of Syy in this range is relatively stable. When H = 0 m, due to the thick weak interlayer, the soft rock around the blast hole is crushed. At this time, the stress wave has been largely consumed, so it is difficult to transmit to the hard rock on both sides, making it in a high stress state, and even cracking the hard rock. Therefore, in this case, the Syy of the rock mass on both sides of the hard rock is small. When 0.5 < H ≤ 3.5 m, it is basically consistent with the above conclusion when H_S_ ≤ 0.3 m.

As shown in Fig. 13b, when the thickness of weak interlayer (H_S_) is constant, Syy shows an overall downward trend with the increase of the distance between blast hole and structural plane (H), and the overall law of the curve is basically consistent with Fig. 13a. When the weak interlayer is within the fourfold crushing zone, the hard rocks on both sides are in a high stress state, and Syy shows an overall attenuation trend with the increase of H. When the weak interlayer is outside the fourfold crushing area, the Syy of the rock masses on both sides is relatively low and basically stable, and the Syy decreases slowly with the increase of H.

### Evolution of rock mass energy fields

**Figure 14:**
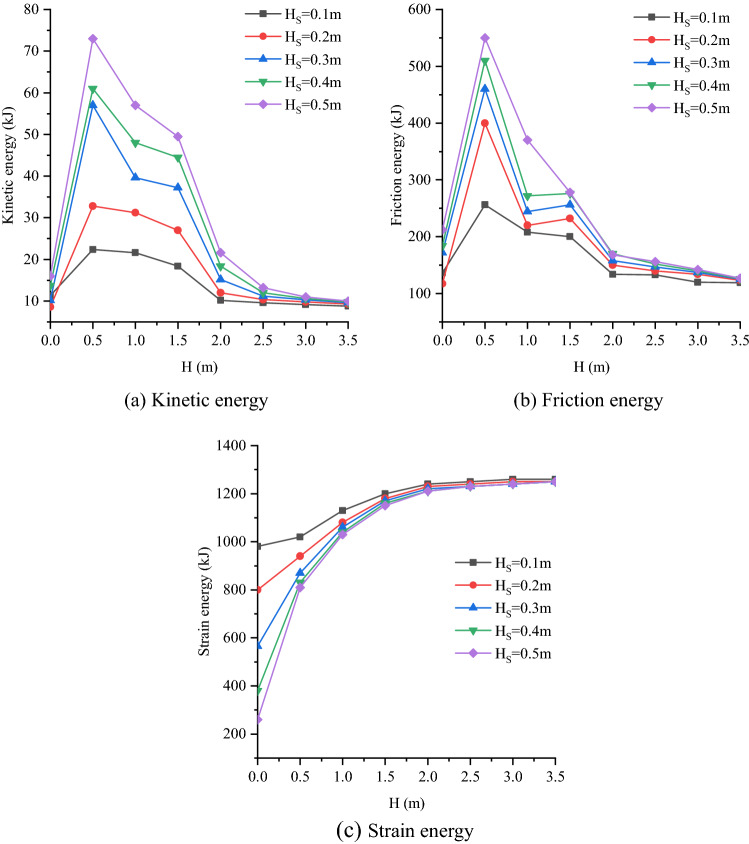
Peak energy variation curve.

In order to explore the influence of the position and thickness of weak interlayer on rock mass energy, this paper analyzes the blasting results from the perspective of kinetic energy, friction energy and strain energy. Three energy calculation methods (formulas 9, 10 and 11) are given in the particle flow code (PFC^2D^). The energy evolution curves of rock mass under different soft rock thickness (H_S_) and different position (H) can be obtained through the energy records in the software (Fig. 14).

Kinetic energy (*E*_*K*_) calculation method:

9 $$E_{K} { = }\sum\limits_{i = 1}^{n} {\frac{1}{2}{\text{m}}_{i} v_{i}^{2} }$$

where m_*i*_ is the mass of the particle, *v*_*i*_ is the velocity of the particle and n is the total number of particles.

Friction energy (*E*_*F*_) calculation method:

10 $$E_{F} = - {\text{F}}^{{\text{d}}} \cdot \left( {\begin{array}{*{20}c} \cdot \\ \delta \\ \end{array} t} \right)$$

where F^d^ is the dashpot force, $$\begin{array}{*{20}c} \cdot \\ \delta \\ \end{array}$$ is the relative translation velocity and *t* is the during time.

Strain energy (*E*_*S*_) calculation method:

11 $$E_{S} = \frac{1}{2}\left( {\frac{{F_{n}^{2} }}{{k_{n} A}} + \frac{{\parallel {\text{F}}_{{\text{s}}} \parallel^{2} }}{{k_{s} A}} + \frac{{M_{t}^{2} }}{{k_{s} J}} + \frac{{\parallel {\text{M}}_{{\text{b}}} \parallel^{2} }}{{k_{n} I}}} \right)$$

where *k*_*n*_ is the normal stiffness, *k*_*s*_ is the shear stiffness, A is the cross-sectional area, *I* is the moment of inertia of the parallel bond cross-section, *J* is the polar moment of inertia of the parallel bond cross-section, *F*_*n*_ is the parallel-bonded normal force, F_s_ is the parallel-bonded shear force, *M*_*t*_ is the parallel-bonded twisting moment (2D model: *M*_*t*_ = 0) and M_b_ is the parallel-bonded bending moment.

As shown in Fig. 14a and b, when H_S_ is constant, the peak kinetic energy and peak friction energy of rock mass samples can be divided into three parts with the increase of H, respectively. When H < 0.5 m, it is the growth section, and the two energy peaks increase rapidly with the increase of H. When 0.5 ≤ H ≤ 2.0 m, it is the rapid decline section, and the two energy peaks decrease rapidly with the increase of H. When H > 2.0 m, it is the slow decline section, and the two energy peaks decrease slowly with the increase of H. When the position of weak interlayer and blast hole (H) is within the range of one time of crushing area, that is, the growth section curve. When H_S_ increases, the values of the two curves increase as a whole, mainly in that H = 0.5 m. When the position of weak interlayer and blast hole is within the range of 1–4 times of crushing area, that is, the curve of rapidly falling section. Within this range, when H_S_ increases, similarly, the values of the two curves are also increasing as a whole. The influence of H_S_ change on kinetic energy peak and friction energy peak is mainly concentrated in the range of 0.5 ≤ H ≤ 2.0 m. After calculation, within this range, the kinetic energy peak and friction energy peak will increase by about 23 and 13% every 0.1 m increase of H_S_. When the position of weak interlayer and blast hole (H) is certain and outside the fourfold crushing area, that is, the curve of slow descent section. Within this range, the peak kinetic energy and friction energy are less affected by the changes of H and H_S_, and the changes of the two peaks are relatively stable.

As shown in Fig. 14c, when H_S_ is constant, it can be seen that the peak strain energy increases as a whole with the increase of H. According to the variation amplitude of the peak strain energy curve, the curve can be divided into two parts. When H ≤ 2.0 m, the peak value of strain energy increases rapidly with the increase of H. When H > 2.0 m, the peak value of strain energy tends to be flat with the increase of H, the change is small and basically in a stable state. When the position of weak interlayer and blast hole (H) is within the range of 4 times of crushing area, the variation range of strain energy (the difference of strain energy between H = 0.0 m and H = 2.0 m) increases with the increase of H_S._ It is calculated that the peak strain energy will increase by about 218 kJ for every 0.1 m increase of H_S_. When the weak interlayer and blast hole position are outside the fourfold crushing area, the variation range of the peak strain energy is small and stable at about 1240 kJ, and basically does not change with the change of H and H_S_.

## Discussions

According to the single hole blasting test results of different thickness and position of weak interlayer considered in this paper, both factors have an obvious impact on the blasting effect. At the same time, the blasting results can be more controllable by adjusting the initiation position and initiation distance:

1. In the blasting experiment carried out in this paper, the maximum thickness of weak interlayer is 0.5 m, which is far less than the thickness of hard rock. Therefore, it is concluded that it does not represent the condition of large thickness of soft rock. From the perspective of blasting crack effect, when blasting in soft rock and the thickness of weak interlayer is much less than that of hard rock, the crack propagation range is small, and the propagation range will be smaller with the increase of soft interlayer thickness. Therefore, when the weak interlayer is quite thin, blasting in soft rock has little impact on the safety and stability of surrounding rock mass. When blasting in hard rock and the distance between weak interlayer and blast hole is within about twice the radius of crushing area, the damage degree of rock mass around blast hole is higher, and the crack propagation range is larger than that in soft rock. When the blasting is initiated in hard rock and the distance between the weak interlayer and the blast hole is outside the radius of about twice the crushing area, the crack propagation range is the largest, and the crushing degree of the rock mass around the blast hole is slightly weaker than the above two working conditions. And in this case, it has the greatest impact on the safety and stability of the surrounding rock mass.
2. From the stress monitored on both sides of the weak interlayer, when the weak interlayer is within 4 times the radius of the crushing area, the hard rocks on both sides of the weak interlayer are in a high stress state, making the hard rocks on the upper side of the weak interlayer unsafe. When the soft interlayer is outside the radius of 4 times the crushing area, the stress on both sides of the soft interlayer is low, which makes the hard rock on the upper side of the soft interlayer safer.
3. From the perspective of kinetic energy, when the weak interlayer is within the radius of 4 times the crushing area, the overall peak kinetic energy of rock mass samples is high, and the change of peak kinetic energy in this range is also large, which has a great influence on the stability of rock mass samples and surrounding rock. When the weak interlayer is outside the radius of 4 times the crushing area, the overall peak kinetic energy of the rock mass sample is small and stable, which has little impact on the stability of the rock mass sample and surrounding rock.
4. In addition to the influence of weak interlayer with different position and thickness on single hole blasting effect considered in this paper, due to the complexity of the stratum, there may be many factors such as joints or high temperature, which makes it difficult to accurately control the blasting effect. Based on complex strata, further in-depth research on these factors will be carried out in the later stage.

## Conclusions

In this paper, the numerical model of hard rock blasting is established by using particle flow code (PFC^2D^), and the rationality of blasting method is verified. On this basis, the soft rock is introduced into the mode, and the weak interlayer single hole blasting experiment is carried out. From the perspective of blasting crack effect, stress field and energy field, the main conclusions are as follows:

1. Compared with the crack effect of single hole blasting in pure hard rock, when the weak interlayer is located within the radius of about twice the crushing area, the closer the interlayer is to the blast hole, the higher the damage degree of the rock mass around the blast hole. Outside this range, the influence on the blasting effect is slight. When the position of weak interlayer is fixed, its ability to reflect stress wave and aggravate rock mass failure increases with the growth of its thickness. When blasting in hard rock, the number of cracks will increase by about 1–2% under the same cases when the distance between weak interlayer and blast hole decreases by 0.5 m.
2. According to the stress state on the upper and lower sides of the weak interlayer, when the explosion is initiated in the hard rock and the weak interlayer is within about 4 times the radius of the crushing area, the hard rocks on both sides of the weak interlayer are in a high stress state, while outside 4 times the radius of the crushing area, the stress of the hard rocks on both sides is low. In both cases, the stress on both sides of the weak interlayer decreases with the increase of its distance from the blast hole.
3. The influence of the thickness of the weak interlayer on the peak kinetic energy, peak friction energy and peak strain energy is mainly reflected in the range that the interlayer is 4 times the radius of the crushing area from the blast hole. Within this range, the kinetic energy peak and friction energy peak will increase by about 23 and 13% and the strain energy peak will increase by about 218 kJ for every 0.1 m increase in the thickness of the weak interlayer. When the weak interlayer is outside the radius of 4 times the crushing area, the changes of the three energies are relatively stable and are less affected by the position and thickness of the interlayer.

## Data Availability

All data generated or analysed during this study are included in this published article.

## References

[1] Liu C-Z, Zhang J-J, Cui P (2018). Energy evolution and stress response during stress wave prorogation in the intercalation. Yantu Lixue Rock Soil Mech..

[2] Wang W, Li X, Zuo Y (2006). Effects of single joint with nonlinear normal deformation on P-wave propagation. Yanshilixue Yu Gongcheng Xuebao Chin. J. Rock Mech. Eng..

[3] Sun N, Lei M, Zhang Y, Su G, Huang G (2020). A study on the influence of weak interlayer on the propagation process of explosion stress wave. Zhendong yu Chongji J. Vib. Shock.

[4] Qian D, Xinping L, Yongsheng J, Jinshan S (2021). A numerical simulation of blasting stress wave propagation in a jointed rock mass under initial stresses. Appl. Sci..

[5] Babanouri N, Fattahi H (2018). Evaluating orthotropic continuum analysis of stress wave propagation through a jointed rock mass. Bull. Eng. Geol. Environ..

[6] Li H (2016). Numerical modeling of wave transmission across rock masses with nonlinear joints. Rock Mech. Rock Eng..

[7] Resende R, Lamas LN, Lemos JV, Calçada R (2010). Micromechanical modelling of stress waves in rock and rock fractures. Rock Mech. Rock Eng..

[8] Deng XF, Zhu JB, Chen SG, Zhao J (2012). Some fundamental issues and verification of 3DEC in modeling wave propagation in jointed rock masses. Rock Mech. Rock Eng..

[9] Du, J., He, T., Xiong, Y. & Zheng, R. Numerical simulation of damage characteristics of jointed rock under blasting load. In *IOP Conference Series: Earth and Environmental Science*, Vol. 861, 072136 10.1088/1755-1315/861/7/072136 (2021).

[10] Tao J (2021). Thermal-mechanical modelling of rock response and damage evolution during excavation in prestressed geothermal deposits. Int. J. Rock Mech. Min. Sci..

[11] Xu H (2020). A closed-form solution to spherical wave propagation in triaxial stress fields. Int. J. Rock Mech. Min. Sci..

[12] Tao J (2020). Effects of in-situ stresses on dynamic rock responses under blast loading. Mech. Mater..

[13] Tao J (2020). Numerical investigation of blast-induced rock fragmentation. Comput. Geotech..

[14] Guo D-Y, Zhang H-J, Lu P-F, Zhang G-W (2014). Effect of fault on deep-hole cumulative blasting to improve coal bed permeability. Beijing Keji Daxue Xuebao J. Univ. Sci. Technol. Beijing.

[15] Zhu G-A (2016). Dynamic behavior of fault slip induced by stress waves. Shock Vib..

[16] Feng X, Zhang Q, Ali M (2019). Explosion-induced stress wave propagation in interacting fault system: numerical modeling and implications for Chaoyang coal mine. Shock Vib..

[17] Gao K (2021). Coal–rock damage characteristics caused by blasting within a reverse fault and its resultant effects on coal and gas outburst. Sci. Rep..

[18] Feng X (2020). 3D modeling of the influence of a splay fault on controlling the propagation of nonlinear stress waves induced by blast loading. Soil Dyn. Earthq. Eng..

[19] Fan L-M, Yan N, Li N (2006). Dynamic response model for thin soft interlayer considering interbedded reflecting waves. Yanshilixue Yu Gongcheng Xuebao Chin. J. Rock Mech. Eng..

[20] Liu G-W, Song D-Q, Chen Z, Yang J-W (2020). Dynamic response characteristics and failure mechanism of coal slopes with weak intercalated layers under blasting loads. Adv. Civ. Eng..

[21] Nan S (2021). The influence mechanism of the master weak interlayer on bench blasting effect and its evaluation method. Shock. Vib..

[22] Ma C, Zhan H, Yao W, Yu H (2018). Stability and safety criterion of a slope with weak interlayer under blasting vibration. Baozha Yu Chongji Explos. Shock Waves.

[23] Wang G, Wang X, Hu S (2015). A dynamic measurement method of elastic modulus of weak interlayer of rock mass. Chin. J. Rock Mech. Eng..

[24] Duan S-Q (2017). In situ observation of failure mechanisms controlled by rock masses with weak interlayer zones in large underground cavern excavations under high geostress. Rock Mech. Rock Eng..

[25] Song S (2019). Model test study on vibration blasting of large cross-section tunnel with small clearance in horizontal stratified surrounding rock. Tunn. Undergr. Space Technol..

[26] Zhang X, Yang Q, Pei X, Du R (2022). Research on dynamic response of slopes with weak interlayers under mining blasting vibration. Front. Energy Res..

[27] Man J, Huang H, Ai Z, Chen J (2022). Analytical model for tunnel face stability in longitudinally inclined layered rock masses with weak interlayer. Comput. Geotech..

[28] Zhou H, He C (2020). Propagation law of stress wave and cracks in non-penetrating jointed rock mass: A numerical study based on particle flow code. Geotech. Geol. Eng..

[29] Cundall PA, Strack ODL (1979). A discrete numerical model for granular assemblies. Géotechnique.

[30] Yuan W (2018). Numerical study of the impact mechanism of decoupling charge on blasting-enhanced permeability in low-permeability sandstones. Int. J. Rock Mech. Min. Sci..

[31] Kouroussis G, Verlinden O, Conti C (2011). Finite-dynamic model for infinite media: Corrected solution of viscous boundary efficiency. J. Eng. Mech..

[32] China Architecture & Building Press. Numerical simulation technology and application with particle flow code (Pfc5.0), China Architecture & Building Press, Chong Shi, China (2018).

[33] Liangfu X, Qingyang Z, Yongjun Q, Jianhu W, Jiangu Q (2020). Study on evolutionary characteristics of toppling deformation of anti-dip bank slope based on energy field. Sustainability.

